# Artery of Percheron Stroke: A Case Report

**DOI:** 10.15388/Amed.2025.32.1.14

**Published:** 2025-02-18

**Authors:** Milda Navickaitė, Aleksandras Vilionskis, Austėja Dapkutė, Kristina Ryliškienė

**Affiliations:** 1 Faculty of Medicine, Vilnius University, Vilnius, Lithuania; 2 Clinic of Neurology and Neurosurgery, Institute of Clinical Medicine, Faculty of Medicine, Vilnius University, Vilnius, Lithuania

**Keywords:** artery of Percheron, ischemic stroke, thalamic infarct, midbrain, Peršerono arterija, išeminis insultas, gumburo infarktas, vidurinės smegenys

## Abstract

**Background:**

The *Artery of Percheron* (AOP) is a vascular variant supplying both sides of the thalamus, present in up to one-quarter of the general population. AOP occlusion is a rare cause of ischemic stroke, resulting in bilateral thalamic infarction. It typically manifests as altered consciousness, gaze abnormalities, and cognitive impairment. Neuroimaging of AOP stroke is challenging, as head CT is often unremarkable. However, a diagnostic ‘V’ sign can be identified on MRI. AOP stroke is treated as other types of ischemic stroke.

**Case description:**

We present a case of a 61-year-old male with a history of alcohol abuse, diagnosed with ischemic AOP stroke. He presented with sudden loss of consciousness, third nerve palsy, and vertical gaze palsy. MRI revealed bilateral paramedian thalamic infarction with midbrain involvement. Despite conservative treatment, his condition showed minimal improvement, leaving him lethargic and dysarthric. He was discharged to palliative care after two weeks.

**Conclusions:**

AOP infarction, though rare, should be considered in patients with altered consciousness. Early MRI is essential for accurate diagnosis and timely treatment, highlighting the importance of physician awareness of this condition.

## Abstract (it)

**Apžvalga:**

Peršerono arterija – tai gumburo kraujotakos variantas, kai abi gumburo puses maitina viena arterija. Ji gali būti aptinkama iki 25 proc. bendrosios populiacijos. Peršerono arterijos okliuzija yra labai reta išeminio insulto priežastis, sukelianti abipusį gumburo infarktą. Šis insultas paprastai pasireiškia sąmonės, akių judesių ir pažinimo funkcijų sutrikimu. Peršerono arterijos infarkto vaizdinimas yra sudėtingas, nes galvos KT dažnai būna be pakitimų. Tačiau galvos MRT galima matyti diagnostinį „V“ ženklą. Peršerono arterijos insultas gydomas kaip ir bet kurios kitos rūšies išeminis insultas.

**Atvejo aprašymas:**

Į ligoninę paguldytas 61 metų vyras, vartojęs alkoholį, kuriam pasireiškė staigus sąmonės netekimas, trečiojo galvinio nervo paralyžius ir vertikalaus žvilgsnio paralyžius. MRT nustatytas abipusis paramedialinis gumburo infarktas su vidurinių smegenų pažeidimu. Pacientui diagnozuotas išeminis Peršerono arterijos insultas. Nepaisant konservatyvaus gydymo, pacientas išliko vangus ir dizartriškas. Po dviejų savaičių buvo išrašytas paliatyviajai slaugai.

**Išvados:**

Nors Peršerono arterijos infarktas pasitaiko retai, apie jį reikėtų pagalvoti tiriant pacientus, kurių sąmonė būna sutrikusi. Ankstyvas MRT tyrimas yra labai svarbus šio tipo išeminio insulto diagnostikai ir gydymui laiku.

## Introduction

The blood supply to the thalamus is complex and greatly varies among humans. The *Artery of Percheron* (AOP) represents a vascular variant of the perforating arteries supplying the thalamus and midbrain, characterised by a single artery providing bilateral blood supply to the thalamus. It was first described by Gérard Percheron in 1973 and is estimated to occur in up to 26% of the general population [1,2]. Occlusion of AOP results in bilateral thalamic infarction and accounts for 4–18% of thalamic strokes [3]. However, studies indicate that this type of ischemic stroke constitutes only 0.27 and 0.8% of all cerebral infarcts, highlighting the rarity of AOP territory strokes [4]. Due to the extensive role of thalamus in various neurological functions, AOP stroke can present with diverse clinical manifestations. The most common symptoms include disturbances of consciousness, memory impairment, and oculomotor dysfunction [5]. The unusual and unpredictable clinical presentation, coupled with difficult visualisation on primary imaging, make early diagnosis of AOP occlusion particularly challenging. Nevertheless, if detected in a timely manner, AOP territory ischemic stroke can be treated with thrombolysis, potentially preventing further neurological deficits [6].

In this report, we present the case of a 61-year-old male who experienced an acute loss of consciousness and was later diagnosed with bilateral thalamic and rostral midbrain stroke caused by AOP occlusion. His clinical presentation was atypical for acute ischemic stroke, complicating the diagnostic process and necessitating differential diagnosis to include other neurological pathologies.

## Case report

A 61-year-old male with a medical history of excessive alcohol consumption was admitted to the emergency department with a sudden loss of consciousness. According to his family, he was last seen conscious 7 hours ago drinking alcohol with a neighbour. On admission, his blood pressure was markedly elevated (223/112 mmHg), he was bradycardic with a pulse rate of 51 beats per minute and normoxic on room air. His Glasgow Coma Scale was 8/15 (eye 1, verbal 2, motor 5), and his *National Institutes of Health Stroke Scale* (NIHSS) score for category 1a (‘Level of consciousness’) was 2 (‘Requires repeated stimulation to arouse’). He responded symmetrically and proportionally to painful stimuli with the movement of all four limbs and face grimacing.

**Figure 1:**
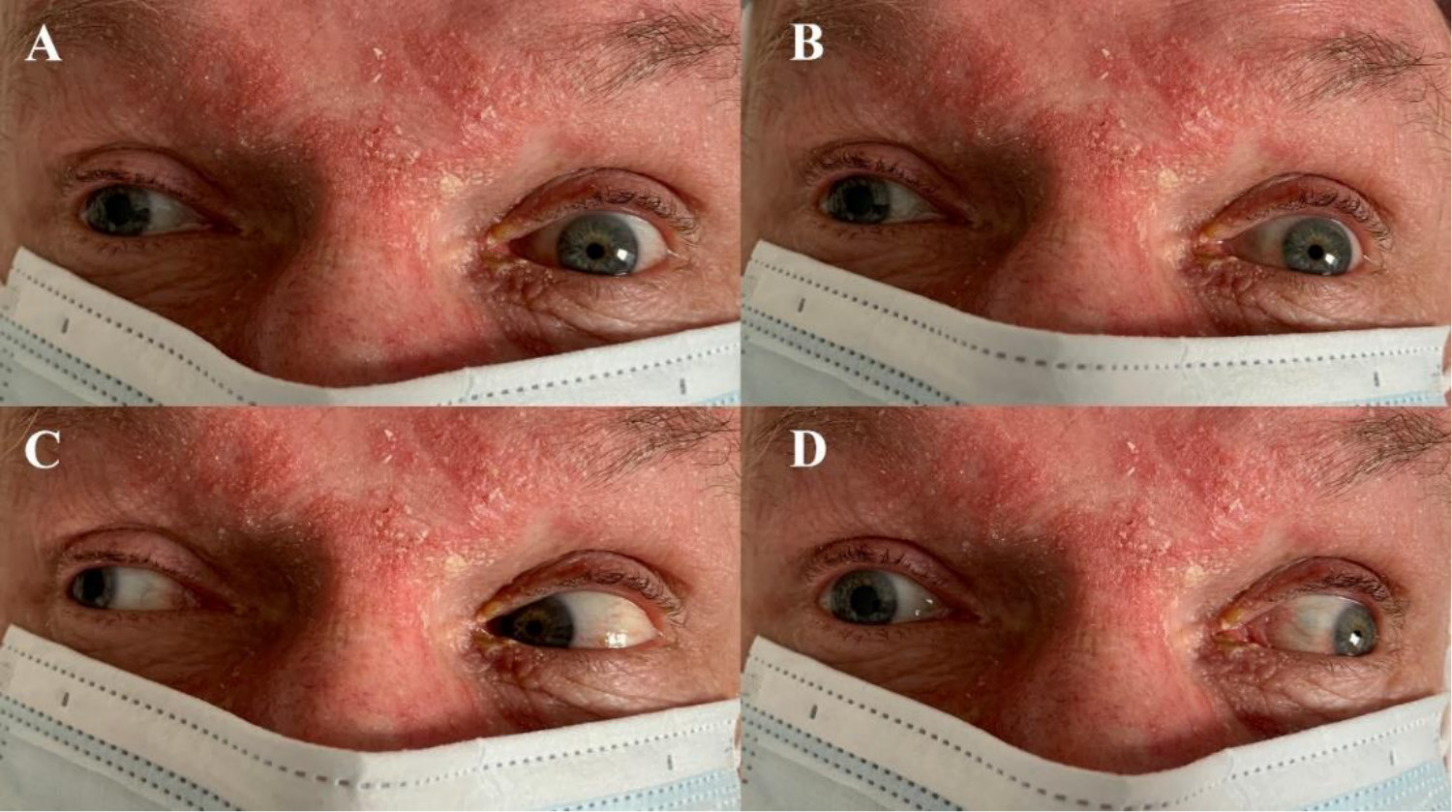
Oculomotor dysfunction in the patient with AOP stroke. (A) Eyes in primary position; (B) Upward gaze; (C) Gaze to the right; (D) Gaze to the left

Cranial nerve examination revealed right-sided third nerve palsy, absent pupillary light reflex bilaterally and anisocoria (left pupil: 2 mm; right pupil: 5 mm). Divergent strabismus was noted, with the right eye deviation outward and downward. The right eye medial gaze was impaired, accompanied by bilateral upward and downward gaze palsy and right-sided ptosis (Figure 1). Further neurological examination revealed no facial asymmetry, normal muscle tone and reflexes in all limbs, and no pathological reflexes. Conventional stroke laboratory panels showed no abnormalities. A blood alcohol test was negative.

**Figure 2:**
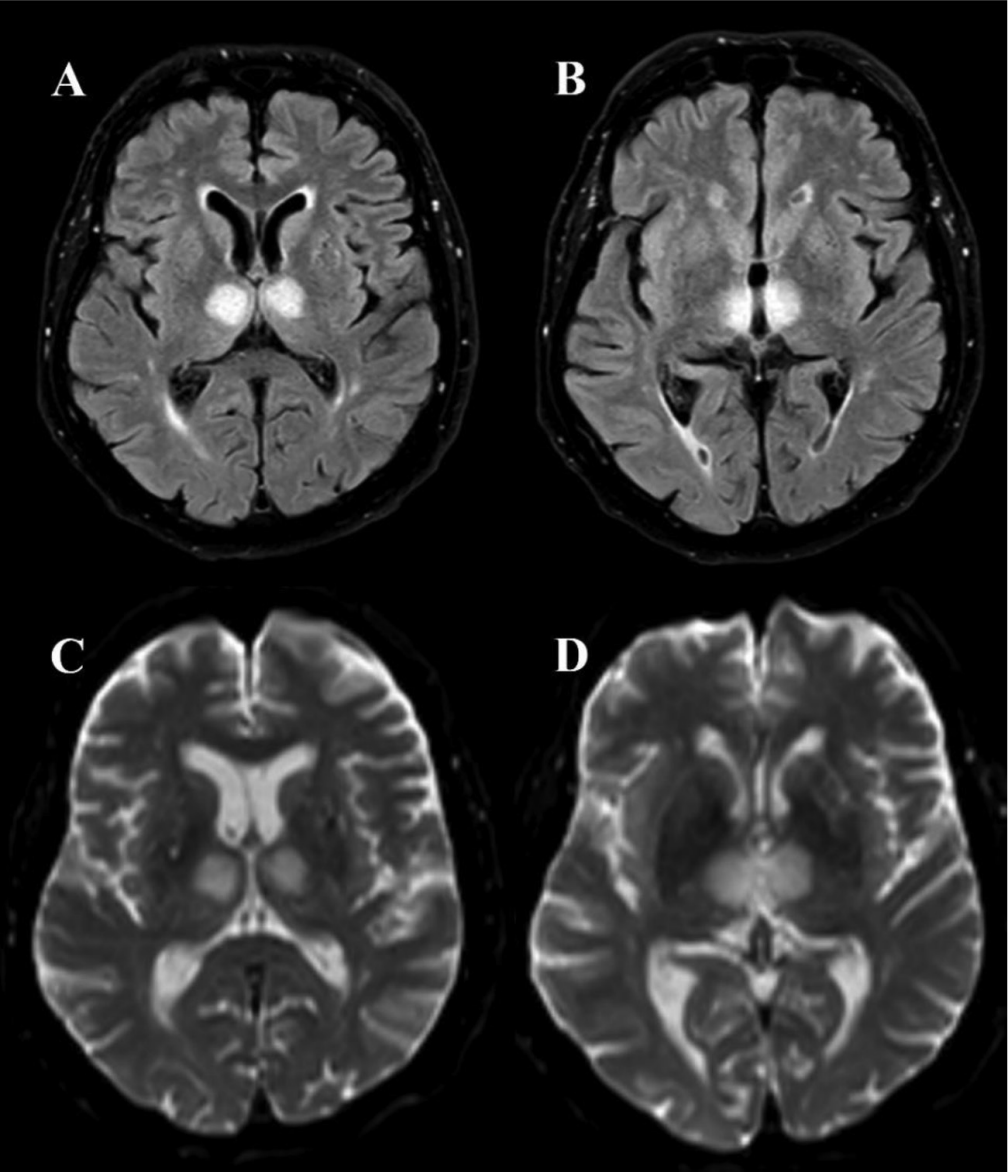
Axial MRI images: FLAIR (A and B) and DWI (C and D) sequences, showing hyperintense bilateral paramedian thalamic signal. MRI, magnetic resonance imaging; FLAIR, fluid attenuated inversion recovery; DWI, diffusion weighted imaging

An urgent head *Computed Tomography* (CT) revealed hypodensity in the bilateral thalami. *CT Angiography* (CTA) was unremarkable. Empiric treatment for possible Wernicke encephalopathy (intravenous vitamin B complex) and hypertension (magnesium sulphate solution and sublingual nitroglycerin) was administered but proved ineffective. *Magnetic Resonance Imaging* (MRI) performed the following day revealed an acute infarction in the bilateral paramedian thalami and medial rostral midbrain, consistent with AOP territory ischemia (Figure 2). As the therapeutic window for thrombolysis had passed, conservative treatment with antiplatelet medication, hypertension control, and statins was initiated.

During his hospitalisation, the patient gradually regained consciousness but remained lethargic and unable to care for himself. His GCS improved to 11/15 (eye 3, verbal 2, motor 6), and his NIHSS score for category 1a returned to 0. However, severe dysarthria persisted, and his neuro-ophthalmological examination showed no improvement, with persistent right-sided third nerve palsy, anisocoria, and bilateral vertical gaze palsy. Due to the lack of neurological improvement, the patient was discharged to a palliative care facility after two weeks.

## Literature review and discussion

The thalamus and midbrain receive their arterial supply from the perforating branches of the P1 and P2 segments of the posterior cerebral artery (PCA) and the posterior communicating artery (PComA). Four territories of thalamic vascularization are recognised: anterior, paramedian, posterior, and inferolateral. Each territory contains distinct nuclei, and stroke symptoms vary depending on the nuclei affected.

Strokes in the tuberothalamic or anterior territories primarily impact arousal, orientation, memory, and executive function. Paramedian or thalamosubthalamic infarctions, particularly when bilateral, lead to reduced arousal and impairments in learning and memory. Lesions in the posterior territory result in visual field deficits, variable sensory loss, weakness, dystonia, and tremors. Strokes in the inferolateral territory cause contralateral sensory loss, hemiparesis, and hemiataxia [7].

The paramedian area is predominantly supplied by thalamoperforating arteries, originating from the P1 segment of the PCA [8]. One of the most widely adopted classification systems for thalamic vascularization was introduced by Percheron in 1977, describing four anatomical variants of the origin of the perforating vessels (Table 1) [9].

**Table 1:** Classification of thalamoperforating arteries anatomic variants [2,9]

Type of anatomic variant	Description	Prevalence, %
Type I	Arteries arise symmetrically and separately from the right and left PCAs, supplying the ipsilateral thalamus	21
Type IIa	Multiple arteries originate directly from only one of the PCAs	9
Type IIb (AOP)	Arteries arise from a single unilateral trunk, which itself originates from one of the PCAs	26
Type III	Arteries arise from a shared arcade artery that bridges the P1 segments of left and right PCAs	17

The primary risk factors for AOP occlusion aligns with the common cardiovascular risk factors, including arterial hypertension, smoking, diabetes mellitus, dyslipidaemia, atrial fibrillation, and a history of stroke or transient ischemic attack [5]. Small vessel disease (33–40%), cardioembolism (20–33%), large artery diseases (13–22%), and other causes such as malignancy-related hypercoagulability, protein C resistance, or idiopathic aetiologies (13–23%) are the predominant underlying mechanisms for AOP territory infarction [5,10,11]. In our case, a definite aetiology was not identified, however, the patient’s history of alcohol abuse and hypertension were recognised as significant risk factors.

The anatomy and physiology of the thalamus are particularly intricate, therefore, AOP occlusion can manifest as complex clinical syndromes depending on the sub-structures and nuclei involved. The most common clinical presentations are summarised in Table 2. Our patient exhibited the most frequent symptom – an abrupt, transient change in consciousness. He also presented with bilateral vertical gaze palsy, likely attributable to midbrain ischemia, as suggested in the literature [10,12–14]. However, in this case, oculomotor nerve dysfunction was limited to the right side, indicating greater ischemia on the right. Cognitive functions, speech, and memory could not be evaluated upon admission due to the patient’s unconscious state. However, during hospitalization, severe dysarthria became apparent.

**Table 2:** Clinical manifestations of AOP stroke [5,10,12,13,15–17]

Presentation	Symptoms	Prevalence	Structures involved
Changes in level of consciousness	Somnolence, stupor, coma	73% [5] – 94.2% [10,12]	Intralaminar nuclei of the thalamus
Gaze abnormalities	Horizontal and/or vertical gaze paresis, oculomotor nerve palsy, diplopia	53% [12] – 83% [10]	Midbrain tegmentum, rostral interstitial nucleus of the medial longitudinal fasciculus, interstitial nucleus of Cajal, oculomotor nucleus
Cognitive impairment, behaviour changes	Disorientation, confusion, agitation, apathy, disinhibition, memory deficits, pseudobulbar affect	42% (disorientation) [5]; 24% (anterograde amnesia) [12]	Paramedian territory, anterior part of the thalamus, hippocampus
Speech abnormalities	Dysarthria, aphasia	55% [10]	Mediodorsal and intralaminar nuclei
Motor dysfunction	Hemiplegia, extrapyramidal features, cerebellar ataxia	33% (motor deficits) [10]; 22% (cerebellar ataxia) [10]; 12% (extrapyramidal features) [12]	Ventroanterior and ventrolateral thalamic nuclei
Other	Headache, tremors, seizures, hyperthermia	18% (headache) [12]	N/A

Acute AOP infarction presents not only as a clinical challenge, but also as a difficult imaging problem. Neuroimaging plays a crucial role in the diagnosis, treatment, and prognosis of cerebral infarction, especially during the hyperacute phase. However, a definitive diagnosis of AOP stroke is often delayed, as imaging signs of ischemic infarction in the thalamus and midbrain become visible only after the hyperacute phase [15]. The diagnostic process typically begins with a non-contrast head CT in patients presenting with acute neurological symptoms, primarily to differentiate between cerebral haemorrhage and ischemia [18] as was the case with our patient. In our case, CT scan revealed bithalamic hypodensity, which has been previously associated with AOP occlusion in the literature [15,19]. Although CT may show bithalamic hypodensity during the acute phase, its sensitivity in detecting AOP ischemic stroke is limited, with scans remaining normal in up to 50% of cases [7,10]. For approximately one-third of patients, CT findings only become informative 2–5 hours after the stroke onset [12]. While CT is more widely accessible and often performed in hyperacute settings, MRI is a more specific imaging modality for the diagnosis of AOP infarction and should be conducted either as the primary or secondary imaging study [20]. To detect AOP infarct in early stages, FLAIR and DWI sequences are considered the gold standard, with DWI having a sensitivity of 100% [10,19]. In typical cases of AOP stroke during the acute phase, brain MRI commonly reveals symmetrical long T1 and T2 signals, a high bithalamic paramedian FLAIR signal, and an elevated DWI signal [15]. A study by Lazzaro et al. (2010) described four distinct MRI patterns in AOP occlusion. The most common patterns were bilateral paramedian thalamic infarction with rostral midbrain involvement (43%) and isolated bilateral paramedian thalamic infarction (38%). Less common patterns included bilateral paramedian and anterior thalamic infarction with midbrain involvement (14%) and bilateral paramedian and anterior thalamic infarction without midbrain involvement (5%) [20]. In the same study, 67% of patients with AOP stroke demonstrated the ‘V’ sign, a hyperintense signal on axial FLAIR and DWI sequences along the pial surface of the midbrain, particularly in the interpeduncular fossa. Recognizing this distinct V-shaped hyperintense signal can aid in the diagnosis of Percheron artery infarction [20]. In our patient, MRI revealed a rare pattern of bilateral paramedian lesions with anterior thalamic and midbrain involvement. Additionally, CT perfusion may be useful in diagnosing stroke as it visualises the infarction core and salvageable penumbra. However, its sensitivity in acute AOP infarction remains uncertain, as CT perfusion is primarily used in medium- and large-vessel occlusions [5]. Furthermore, the diagnostic accuracy of CT perfusion is reduced at the level of the midbrain due to artefacts [4,19]. Lastly, non-invasive angiographic techniques such as head CTA or MRA are often insufficient for diagnosing AOP occlusion, as the artery of Percheron is too small to visualise. This limitation was also evident in our patient [8].

It is important to note that while bilateral thalamic lesions are uncommon, AOP occlusion is not the only plausible cause. The differential diagnosis primarily includes vascular and metabolic disorders, as outlined in Table 3.

**Table 3:** Main differential diagnoses of the case [14,15,21]

Disease	Presentation and radiological features
Vascular disorders	
Top of the basilar syndrome	Bilateral thalamic infarction with additional infarcts in the superior cerebellar artery and posterior cerebral artery territories.
Deep cerebral venous thrombosis	Bilateral thalamic infarction with basal ganglia involvement. CT: hyperdense vein. MRI: T1 hyperintensity from a venous clot.
Metabolic disorders	
Wernicke encephalopathy	Ataxia, loss of consciousness, abnormal ocular movements; associated with thiamine deficiency and excessive alcohol consumption. MRI: T2 hyperintensity in the thalamus, periaqueductal grey matter, tectal plate, dorsal medulla, and mammillary bodies.
Osmotic myelinolysis	Altered mental state, loss of consciousness, ataxia, spastic quadriparesis, dysphagia, dysarthria; results from the rapid correction of hyponatremia. MRI: T2 hyperintensity in the thalamus, the central pons, basal ganglia, and white matter.

Differential diagnoses should also include infections, neoplasms, cerebral lupus, and Wilson’s disease [22,23]. For our patient, these conditions were excluded based on clinical history, symptoms, and, most importantly, neuroimaging findings.

Accurate detection of an AOP occlusion is essential, as the quality of treatment for acute ischemic stroke depends on factors such as timing, the anatomical location of the lesion, and contraindications to thrombolytics. As with other ischaemic strokes, thrombolysis is the most effective treatment if the patient presents within the therapeutic window of 4.5 hours [6]. In contrast, mechanical thrombectomy, commonly used for other cerebral arterial occlusions, is rarely considered an option for AOP strokes due to the small diameter of the artery and difficult visualisation [22]. Antiplatelet therapy should be initiated within 24–48 hours to prevent stroke recurrence. The treatment regimen should also include risk factor modifications such as blood pressure and glycaemic control, smoking cessation and other lifestyle changes [6,24].

AOP stroke generally has a favourable prognosis if diagnosed promptly and treated appropriately. According to Arauz et al., 67% of patients with bilateral paramedian thalamic infarct achieved functional independence, indicated by a Modified Rankin Scale score of less than 2. However, if the rostral midbrain is involved, only 25% of patients achieve a favourable outcome [11]. This highlights the poorer prognosis for patients with additional midbrain infarction compared to those without. Poor outcomes are also associated with delayed diagnosis, as was the case for our patient, who was admitted to the hospital outside the therapeutic window. Many AOP infarction diagnoses are delayed due to several factors. One reason is the highly variable clinical presentation of AOP stroke, which lacks classic stroke symptoms. According to Ikramuddin et al. (2024), the median time to hospital admission for patients with AOP stroke is approximately 13 hours – which is more than double the median time for the general stroke population. Furthermore, only 58% of AOP stroke patients are diagnosed with ischemic stroke at the time of admission [5]. Another challenge is the difficulty of imaging AOP occlusions with CT or MRI during the early stages. Lastly, the rarity of AOP strokes reduces physician awareness of this diagnosis, further contributing to delays [23].

## Conclusions

Although AOP is a rare anatomical variant of thalamic vascularisation, its territory infarct should be considered in patients presenting with generalised neurological symptoms and disturbance of consciousness. Diagnosing AOP occlusion is often challenging due to its inconsistent and diverse clinical manifestation, reflecting the wide range of thalamic functions. Acute CT imaging is frequently unremarkable in such cases, underscoring the importance of early MRI for timely diagnosis and initiation of treatment. This case highlights the need to include AOP infarction and other thalamic pathologies in the differential diagnosis for patients with impaired consciousness, particularly those with other neurological deficits, such as oculomotor or speech disorders. Given the rarity of AOP occlusions, we aim to raise awareness among physicians about this condition to facilitate earlier recognition and management.
